# League of Remembrance

**Published:** 1923-12

**Authors:** 


### THE LEAGUE OF REMEMBRANCE.

THE Second Annual General Meeting of the League of Remembrance has been held at 1, Marlborough Gate, when Princess Beatrice, President of the League, presided. Mr. John Murray, C.V.O., Chairman of the League, referred to the great success which followed the publication of an article by Ian Hay, entitled "An Ideal Organisation," which sympathetically explained the work of the League and resulted in direct donations of about £500, as well as a considerable addition to the membership. He explained that the League, which undertakes the making of garments, surgical dressings, etc., for hospitals and Welfare Centres, has been asked to make more elaborate dressings and garments than at the beginning of its career, and had, for example, made 169 infants' garments for the new Maternity Ward of St. George's Hospital. The demand for the work of the League was firmly established, and he would like to see it acting as a centre for the preparation of those articles so constantly needed by voluntary and charitable institutions throughout the country. Mr. Gibson, the Hon. Treasurer, stated that the cost of running the League is about £2,000 a year, and that as subscriptions and other sources of income produced about £1,550, a further £450 per annum was needed to put it upon a business footing. Mrs. Gibson, the Hon. General Manager, spoke of the real necessity for the League possessing its own premises, and said that the General Committee hoped shortly to place before the members some practicable scheme whereby a substantial sum could be collected in order to purchase a suitable house for offices and workrooms. She hoped that the membership of the League and its consequent power to do good might steadily increase in years to come, so that future generations might regard it as a living and ever present memorial of the Great War.

---

**Insulin Available For All.**

Insulin (Reuter says) is now being produced at Indianapolis in quantities sufficient to meet the world's needs at a price intended to make it available to the poorest sufferers.

